# Respiration-Locking of Olfactory Receptor and Projection Neurons in the Mouse Olfactory Bulb and Its Modulation by Brain State

**DOI:** 10.3389/fncel.2020.00220

**Published:** 2020-07-16

**Authors:** Tobias Ackels, Rebecca Jordan, Andreas T. Schaefer, Izumi Fukunaga

**Affiliations:** ^1^Neurophysiology of Behaviour Laboratory, The Francis Crick Institute, London, United Kingdom; ^2^Department of Neuroscience, Physiology and Pharmacology, University College London, London, United Kingdom; ^3^Sensory and Behavioural Neuroscience Unit, Okinawa Institute of Science and Technology Graduate University, Okinawa, Japan

**Keywords:** olfaction, temporal coding, olfactory bulb, imaging, electrophysiology, active sampling

## Abstract

For sensory systems of the brain, the dynamics of an animal’s own sampling behavior has a direct consequence on ensuing computations. This is particularly the case for mammalian olfaction, where a rhythmic flow of air over the nasal epithelium entrains activity in olfactory system neurons in a phenomenon known as sniff-locking. Parameters of sniffing can, however, change drastically with brain states. Coupled to the fact that different observation methods have different kinetics, consensus on the sniff-locking properties of neurons is lacking. To address this, we investigated the sniff-related activity of olfactory sensory neurons (OSNs), as well as the principal neurons of the olfactory bulb (OB), using 2-photon calcium imaging and intracellular whole-cell patch-clamp recordings *in vivo*, both in anesthetized and awake mice. Our results indicate that OSNs and OB output neurons lock robustly to the sniff rhythm, but with a slight temporal shift between behavioral states. We also observed a slight delay between methods. Further, the divergent sniff-locking by tufted cells (TCs) and mitral cells (MCs) in the absence of odor can be used to determine the cell type reliably using a simple linear classifier. Using this classification on datasets where morphological identification is unavailable, we find that MCs use a wider range of temporal shifts to encode odors than previously thought, while TCs have a constrained timing of activation due to an early-onset hyperpolarization. We conclude that the sniff rhythm serves as a fundamental rhythm but its impact on odor encoding depends on cell type, and this difference is accentuated in awake mice.

## Introduction

Features of the world are encoded in the brain as the activity of neurons. A fundamental challenge for sensory neuroscience is to understand the nature of this representation, as well as the mechanisms by which it is formed. Transformation and extraction of sensory information into neural activity, or sensory encoding, occurs both in temporal and spatial dimensions (Smith, 2008; Panzeri et al., 2017). Of the temporal characteristics, cyclic or rhythmical activity is observed ubiquitously across the brain. This may emerge as a network phenomenon, as a population of neurons interacting (Singer, 1999; Buzsáki and Draguhn, 2004), but often in sensory systems, it is a direct consequence of animals’ sampling behaviors (Diamond et al., 2008; Schroeder et al., 2010).

In olfaction, the arrival of volatile stimuli is driven by changes in the chest cavity volume. At rest, this is due to breathing that generates rhythmic intakes of air, bringing pulsatile samples of air into the nasal epithelium. In anesthetized rodents, this is the only mode of rhythmic air intake and occurs at 2–4 Hz. In the awake state, in addition to this basic respiratory rhythm, animals may generate active sampling behavior, which ranges from 2–12 Hz depending on the behavioral state (Welker, 1964; Wachowiak, 2011). For simplicity, here, we refer to all modes of rhythmic air intake as sniffing.

Even in the absence of odors, the rhythmic movement of air is thought to cause activity locked to this rhythm in olfactory sensory neurons (OSNs), at least partially due to mechanosenzation (Grosmaitre et al., 2007; Connelly et al., 2015). This forms the basis of the fundamental rhythm that shapes much of the activity within early stages of olfactory processing (Adrian, 1950; Kay and Laurent, 1999; Cang and Isaacson, 2003; Margrie and Schaefer, 2003; Carey et al., 2009; Cury and Uchida, 2010; Shusterman et al., 2011; Iwata et al., 2017; Moran et al., 2019). This is particularly the case for the principal neurons of the olfactory bulb (OB), mitral cells (MCs) and tufted cells (TCs), that convey the result of the computation of this region. While similar, these two neuron types differ in physiological and anatomical traits. The two cell types can be distinguished by their soma location, distribution of lateral dendrites (Haberly and Price, 1977; Mori et al., 1983; Orona et al., 1984; Fukunaga et al., 2012; Igarashi et al., 2012), and projection targets (Haberly and Price, 1977; Nagayama et al., 2010; Igarashi et al., 2012). Even though both cell types receive excitatory synaptic inputs from the OSNs (Shepherd, 2004), MCs and TCs lock differently to the sniff-rhythm (Fukunaga et al., 2012; Igarashi et al., 2012; Phillips et al., 2012; Jordan et al., 2018a). This difference in sniff-locking in the absence of odors has been suggested as a physiological signature of cell identity, and further, may be the basis of distinct olfactory encoding that the two cell types use (Fukunaga et al., 2012).

To date, sniff-coupled activity in MCs and TCs has been described using both electrophysiological (Adrian, 1950; Cang and Isaacson, 2003; Margrie and Schaefer, 2003; Carey and Wachowiak, 2011; Shusterman et al., 2011; Smear et al., 2011; Fukunaga et al., 2012, 2014; Igarashi et al., 2012; Díaz-quesada et al., 2018; Jordan et al., 2018a,b) and imaging techniques (Iwata et al., 2017; Short and Wachowiak, 2019; Eiting and Wachowiak, 2020) in a variety of brain states. However, it is unclear how sniff-locking under wide-ranging protocols relates to each other, especially given that parameters of sniff patterns, or inputs to the olfactory system, can change drastically between anesthetized and awake states (Welker, 1964; Jessberger et al., 2016). Further, the reliability of cell-type determination from baseline sniff-locking, and what this means for MC vs. TC encoding of odors, needs to be examined. To this end, here, we describe and compare sniff-locking of OSNs, as well as MCs and TCs, using whole-cell patch-clamp recordings and two-photon microscopy in both awake and anesthetized animals. We then apply cell-type identification from baseline sniff-locking in both states to analyze how MCs and TCs encode olfactory information.

## Materials and Methods

All mice used were bred in-house. C57BL/6 Jax males aged between 5 and 12 weeks or Tbet-cre (Haddad et al., 2013; Jax stock #024507) or OMP-cre (Ishii et al., 2004; JAX stock #006668) mice were crossed with a GCaMP6f reporter line (Madisen et al., 2015; JAX stock #028865) to drive GCaMP6f expression. All animal experiments were approved by the ethics panel of the Francis Crick Institute and the OIST Graduate University, and according to the guidelines of the German animal welfare law.

### Surgical Procedures

Acute surgery for imaging: Aseptic surgical technique was applied. Mice were anesthetized using a mixture of fentanyl/midazolam/medetomidine (0.05 mg·kg^−1^, 5 mg·kg^−1^, 0.5 mg·kg^−1^ respectively; 11 mice) or ketamine/xylazine (100 mg·kg^−1^/20 mg·kg^−1^ for induction, 10 mg·kg^−1^ for maintenance; three mice). The depth of anesthesia was monitored throughout by testing the toe-pinch reflex. The fur over the skull and at the base of the neck was shaved and the exposed skin sterilized with a 1% chlorhexidine solution. Mice were then placed on a thermoregulator (DC Temperature Controller, FHC, ME, USA) heat pad to maintain the body temperature at 36.5°C with a temperature probe inserted rectally. While on the heat pad, the head of the animal was held in place with a set of ear bars. The scalp was incised and pulled away from the skull with four arterial clamps at each corner of the incision. A custom head implant was attached to the base of the skull with medical super glue (Vetbond, 3M, Maplewood, MN, USA), and dental cement (Paladur, Heraeus Kulzer GmbH, Hanau, Germany; Simplex Rapid Liquid, Associated Dental Products Limited, Swindon, UK) was applied around the edges of the implant to ensure firm adhesion to the skull. A craniotomy over the left OB (approximately 2 × 2 mm) was made with a dental drill (Success 40, Osada, Tokyo, Japan) and immersed in artificial cerebrospinal fluid [NaCl (125 mM), KCl (5 mM), HEPES (10 mM), pH adjusted to 7.4 with NaOH, MgSO_4_.7H_2_O (2 mM), CaCl_2_.2H_2_O (2 mM), glucose (10 mM)] before removing the skull with forceps. The dura was then peeled back using fine forceps. A layer of 2% low-melt agarose (Sigma-Adrich, St. Louis, MO, USA) dissolved in the artificial cerebrospinal fluid was applied over the exposed brain surface before placing a 3 mm glass window (borosilicate glass 1.0 thickness) over the craniotomy. The edges of the window were then glued with medical super glue (Vetbond, 3M, Maplewood MN, USA) to the skull.

Recovery surgery for imaging: For implantation of the head-plate, mice were anesthetized with isoflurane in 95% oxygen (5% for induction, 1.5–3% for maintenance). Local (mepivacaine, 0.5% s.c.) and general analgesics (carprofen 5 mg/kg s.c.) were applied immediately at the onset of surgery. For awake imaging, craniotomy and window implantation as above were made at this time. After surgery, animals were allowed to recover with access to a wet diet and monitored daily for 3 days with additional analgesia. After 7–14 days, animals were habituated to the head-fixation situation for at least 15 min on three consecutive days preceding the experiment.

### Imaging

Mice were head-fixed and placed under a two-photon microscope (Denk et al., 1990). Anesthetized mice were maintained on a heating pad to keep the body temperature at 36°C. The microscope (Scientifica Multiphoton VivoScope or custom designed by Independent NeuroScience Services, UK) was coupled with a MaiTai DeepSee laser (Spectra-Physics, Santa Clara, CA, USA) tuned to 940 nm (<30 mW average power on the sample) for imaging. Images (512 × 512 pixels) were acquired with a resonant scanner at a frame rate of 30 Hz using a 16× 0.8 NA water-immersion objective (Nikon).

## Olfactometry

Odors were delivered using a custom-made airflow dilution olfactometer (Fukunaga et al., 2012). Odors (Isoamyl acetate, methyl salicylate, salicylaldehyde, eugenol, cinnamaldehyde, ethyl butyrate, all Sigma-Adrich, St. Louis, MO, USA) were presented at 1–5% saturated vapor. Before each experiment, stimuli were calibrated using a photoionization detector (miniPID, Aurora Scientific, Aurora, ON, Canada) so that the concentration profile closely followed a final valve opening. Odors were presented with a minimum inter-trial interval of 20 s, during which high pressure, clean air was passed through the lines to minimize contamination. The flow rate of exchange air, which flows towards the animals when the final valve is not charged, was matched to the flow rate of odorized air, to minimize the tactile component accompanying the odor stimulus.

### Whole-cell recordings

Surgery for awake recording. The Head plate was implanted 7 days prior, as described above. On the day of recording, mice were anesthetized with isoflurane as above, and carprofen analgesic was injected (5 mg/kg s.c.). A 1-mm-diameter craniotomy was made overlying the right OB, and the dura was removed. A layer of 4% low-melting-point agar was then applied to the surface of the bulb, ~0.5–1 mm thick, to reduce brain movement. Buffer solution mimicking cerebrospinal fluid (125 mM NaCl, 5 mM KCl, 10 mM HEPES, 2 mM MgSO_4_, 2 mM CaCl_2_, 10 mM glucose) was used to fill the recording chamber. The animal would then be transferred to the recording rig, its head fixed above a treadmill, and allowed to wake from anesthesia for 20 min. Whole-cell recordings were then made blindly by descending a 5–7-MΩ borosilicate glass micropipette (Hilgenberg, pulled on a DMZ Universal puller, Zeitz Instruments) filled with the intracellular solution (130 mM KMeSO_4_, 10 mM HEPES, 7 mM KCl, 2 mM ATP-Na, 2 mM ATP-Mg, 0.5 mM GTP, 0.05 mM EGTA, and in some cases 10 mM biocytin; pH adjusted to 7.4 with KOH, osmolarity = 280 mOsm) through the agar and 180 μm into the OB with high pressure. Whole-cell patch-clamp recordings were obtained in a manner described in Margrie et al. (2002). Membrane voltage recording was made in current-clamp mode. Mitral and tufted cells were recognized as those with an input resistance <150 MΩ, a resting membrane potential between –60 and –40 mV, and an afterhyperpolarization (AHP) waveform conforming to MTC phenotype in an independent component analysis performed as detailed in previous studies (Kollo et al., 2014; Jordan et al., 2018a). Data from M/TCs with morphological reconstructions were from Fukunaga et al. (2012) and Jordan et al. (2018a). For whole-cell recordings without morphology, only those that significantly couple to the sniff rhythm (69/83 cells; Fukunaga et al., 2012) were considered. Of these, 55 cells were recorded long enough to allow multiple trials of odor presentations and thus used for analyses.

### Sniff Measurement

The nasal flow was recorded by placing a flow sensor (A3100, Honeywell, NC, USA) externally close to the nostril contralateral to the side of the recording and sampled at 1 kHz. The position of the sensor was manually optimized at the start of each session such that all sniff cycles were captured with a high signal-to-noise ratio.

### Data Analysis

Image pre-processing: Motion correction, segmentation, and trace extraction were performed using the Suite2p package (Pachitariu et al., 2016,^1^). Putative neuronal somata were automatically identified by segmentation and curated manually. Soma and neuropil fluorescence traces were extracted and neuropil fluorescence was subtracted from the corresponding soma trace. Further analysis was performed with custom-written scripts in Matlab. ROIs corresponding to glomeruli were manually delineated based on the mean fluorescence image. Fluorescence signal from all pixels within each ROI was averaged and extracted as time series. ΔF/F = (F − F_0_)/F_0_, where F = raw fluorescence and F_0_ was the median of the fluorescence signal distribution. In the presented data, 37 ROIs came from mice anesthetized with ketamine/xylazine, and 826 ROIs came from mice anesthetized with the fentanyl-based anesthetic. No MCs from ketamine/xylazine anesthesia are included in the study. Sniff-coupling properties under the two anesthetics were largely similar, thus the two datasets were pooled (see Supplementary Figure S1).

Sniff signals were analyzed in custom-written routines in Matlab and built-in functions in Spike2 (CED, UK). Inhalation peaks were detected in Spike2 using cursor functions for peak detection. Inhalation onsets were the point of zero-crossing immediately before the inhalation peak. Detected events were checked visually. Time to peak inhalation time was defined as the time from inhalation onset to inhalation peak. Sniff duration was calculated as the time between subsequent inhalation onsets.

### Inhalation-Triggered Average

Inhalation-triggered membrane potential (Vm) average: action potentials were clipped and for each inhalation onset, Vm during the subsequent 700 ms was collected. This was averaged over all inhalation onsets to obtain the average. For the classifier, the average waveform was down-sampled to 1 kHz and normalized, so the amplitude ranged [0–1]. Peak amplitude was obtained using Matlab’s *findpeaks* function, where the search was constrained for events larger than 0.8 for normalized traces. To determine if the amplitude of peak is due to significant sniff-locking, the amplitude of the average waveform at the peak location was obtained from a non-normalized trace. This was compared against the maximum amplitude of a randomly aligned average. The random time points were obtained by permuting the observed sniff intervals. For inhalation-triggered action potential (AP) histograms, action potentials in the 700 ms were counted in bins (bin *size* = 20 ms for display; 10 ms for classifier). Histogram amplitude was normalized to the range [0–1] for the classifier.

### Aligning to Random Time Points

To generate randomly aligned waveforms, sniff intervals were randomly permuted with the timing of the first inhalation onset also generated to fall within the mean interval duration. Inhalation-triggered and warped averages were obtained as described for observed sniff timing.

### Warping

Subthreshold Vm, AP histogram, and calcium (Ca) signals were interpolated so that for each sniff cycle, time points were “stretched” to run from 0–2π radians (Shusterman et al., 2011), and averaged over all sniff cycles. The sniff cycle was defined from an inhalation onset to the next inhalation onset. The resultant vector was calculated as previously described (Fukunaga et al., 2012). Briefly, at each sniff phase, the amplitude of Vm and calcium transients, as well as the height of the AP histogram, were expressed as the length of the vector pointing in the direction of the sniff cycle phase in polar coordinates. Vectors from all phase points were linearly summed, giving a resultant vector. The direction of this resultant vector is the phase preference. The significance of sniff-coupling was assessed as described previously (Fukunaga et al., 2012).

### Fisher’s Linear Discriminant Analysis

A linear classifier was constructed using the *fitcdiscr* function in Matlab, which implements Fisher’s linear discriminant classifier (Fisher, 1936). This calculates a direction that maximizes between-group variance relative to the within-group variance. Test data is projected on this axis to determine the associated label, using the *predict* function in Matlab. Classifiers were constructed from morphologically identified MCs and TCs using inhalation-triggered average waveforms. Input data were normalized so that the amplitudes of Vm, histogram height and calcium transient of each cell ranged from [0–1]. Vm and calcium transients were interpolated to 1 kHz, while histogram bin size was 10 ms. For the subsequent prediction of cell-types from awake mice, where no morphology was available, average sniff-triggered waveforms were constructed from sniff cycles with duration ranging between 300 and 500 ms.

### Analysis of Odor Responses

t-statistics: For each cell-odor pair, the average firing rate during a 2-s odor presentation and 2 s preceding odor presentation were obtained from each trial, and the overall distribution was compared using Matlab’s *t*-test function. The T-statistic was then used to infer the magnitude of the evoked response.

### Membrane Voltage Change Evoked by Odor Presentation

Action potentials were clipped by interpolating the membrane potential between points before and after each action potential, and the average membrane potential during the 2 s before odor onset was subtracted.

## Results

### Respiration Patterns, and the Input to the Olfactory Bulb, are Influenced by Animal’s State

**Figure 1:**
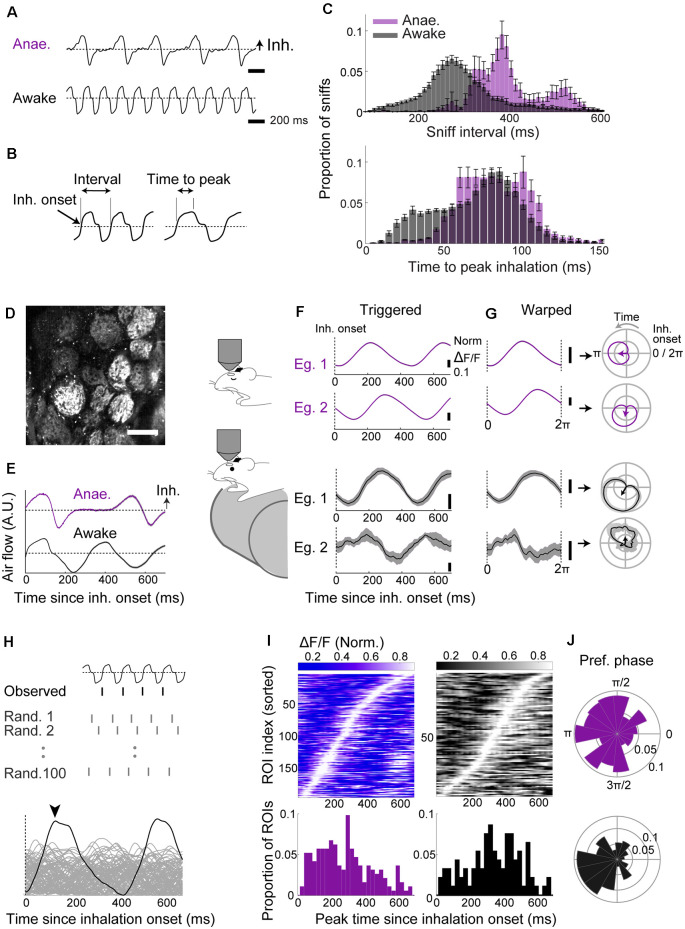
Two-photon imaging reveals robust baseline locking in olfactory sensory neuron (OSN) terminals with a modest temporal shift across behavioral states. **(A–C)** Sniff patterns of anesthetized and awake animals. **(A)** Example flow sensor signals from an anesthetized (top) and awake (bottom) mouse. Inhalation is in the positive direction. The dotted line indicates zero net flow. **(B)** Illustration of parameters: Inhalation onset = time of zero-crossing; sniff interval = from an inhalation onset to the next inhalation onset. Time to peak = latency to peak from the inhalation onset. **(C)** Distribution of sniff parameters for anesthetized (purple; *n* = 21 mice) and awake mice (gray; *n* = 12 mice). **(D)** Example field of view showing GCaMP6f fluorescence from OSN terminals on the dorsal olfactory bulb (OB) surface. Scale bar = 100 μm. **(E)** Examples: inhalation-triggered average sniff waveform for anesthetized (top, purple; 1,090 onsets used) and awake (bottom, black; 3,175 onsets used) mice. Mean ± SEM shown. **(F)** Inhalation-triggered averages for two example glomeruli from anesthetized (top, purple) and awake (bottom, black) mice. **(G)** Left: examples of “warped” averages, from anesthetized (top, purple) and awake (bottom, black) animals. Right: the same “warped” examples plotted in polar coordinates. Sniff phase advances anti-clockwise. **(H)** The approach used to assess the significance of sniff-locking. For each ROI, a sniff-triggered average is obtained by aligning to the observed inhalation onsets or to randomly scattered onsets (100 sets generated). If the peak from alignment to the real onset times (arrowhead) is higher than 95% of the randomly aligned cases, the ROI is said to lock significantly to the sniff rhythm. **(I)** Left top: inhalation-triggered averages from all ROIs that couple significantly to sniff for anesthetized mice; the fluorescence fluctuation is normalized and shown in grayscale. ROI index was sorted by the time of peak. Left, bottom: distribution of peak times concerning the inhalation onset. Right: the same as left panel, but for awake mice. *N* = 215 ROIs, seven mice for anesthetized and *N* = 144 ROIs, four mice for awake. **(J)** Polar histogram of the preferred phase for all significantly sniff-locked glomeruli for anesthetized (top) and awake (bottom) cases.

Sniffing generates the fundamental frequency of olfactory representations, but its pattern can change dynamically, for example, with brain state (Welker, 1964; Wesson et al., 2008b; Jessberger et al., 2016). To characterize this in our experimental setting, we recorded the nasal airflow of head-fixed anesthetized and awake mice by placing a flow sensor unilaterally, close to a nostril (Figure 1A). To anesthetize mice, we injected ketamine/xylazine or fentanyl/midazolam/medetomidine intraperitoneally. For the awake case, mice had been habituated to head-fixation as described in the methods. Consistent with previous studies, sniff parameters (Figure 1B) are different between anesthetized and awake mice, with shorter sniff intervals, and faster intake of air in awake mice (Figure 1C; interval = 301.8 ± 11.0 ms for awake vs. 411.6 ± 9.2 ms for anesthetized; mean ± standard error of the mean; *p* < 0.01; time to peak inhalation = 69.9 ± 1.5 ms for awake vs. 88.9 ± 2 ms for anesthetized; *p* < 0.01, unpaired *t*-test for equal means unpaired *t*-test for equal means; *n* = 21 mice and 12 mice for anesthetized and awake data, respectively).

This drastic difference in sniffing patterns may influence the nature of sniff-locked inputs arriving in the OB (Wesson et al., 2008a). To assess this, using two-photon microscopy, we measured the GCaMP6f signals from OSN axon terminals on the dorsal OB surface in OMP-cre:Rosa-GCaMP6f mice (Figure 1D). Two-hundred and fifteen glomeruli from seven anesthetized mice and 144 glomeruli from four awake, head-fixed mice were analyzed. To assess how glomerular calcium activity locks to sniff cycles, fluorescence signals were collected and expressed first as inhalation-triggered averages (Figure 1F) or, second, aligned, or “warped,” so that the time axis is expressed with respect to the phase of the sniff cycle (Shusterman et al., 2011; Figure 1G). Each sniff cycle here is defined from one inhalation onset to the next. This averaging revealed characteristic peaks locked to inhalation onsets, indicating consistent activation with the sniff rhythm (Figure 1F). To determine if this sniff-locking is statistically significant, we compared the peak amplitude to those derived from randomly permuted sniff intervals (Figure 1H). We assigned glomeruli to be significantly locked if the observed amplitude exceeded at least 95% of randomly generated cases. Similarly, for the warped averages, we compared the length of the resultant vector to those generated by randomly dispersed intervals (Figure 1H; Fukunaga et al., 2012). This revealed that the majority of glomeruli couple to sniffs significantly (90.2% in anesthetized and 64.6% in awake with inhalation-triggered average; 100% and 90.3% with warped alignment).

In awake mice, despite faster air intake, the time of peak fluorescence is shifted to later compared to anesthetized cases (Figure 1I; 282.7 ± 11.6 ms in anesthetized vs. 344.4 ± 16.5 ms in awake; *p* = 0.003, unpaired *t*-test for equal means; 194 and 93 glomeruli for anesthetized and awake states, respectively). Thus, the phase shift between anesthetized and awake (Figure 1J) likely arises as a combination of a change in cycle length and the absolute timing of signal arrival. Overall, the result indicates that OSN input locks tightly in both anesthetized and awake animals, but with a slight temporal shift across the states.

### Olfactory Bulb Projection Neurons Exhibit Diverging Sniff-Locking in Anesthetized Animals

**Figure 2:**
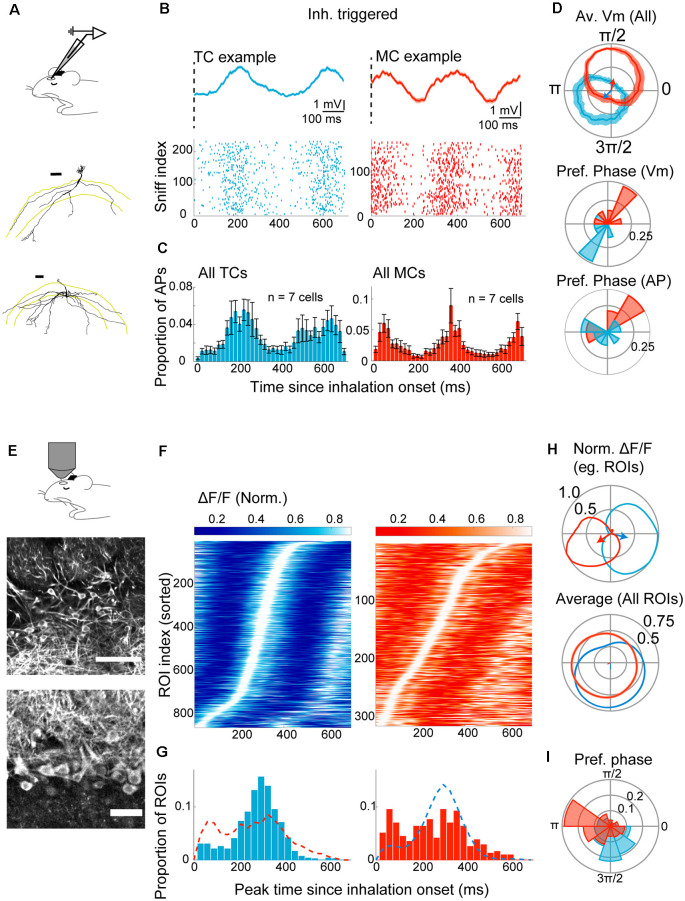
Divergent sniff-locking of tufted cells (TCs) and mitral cells (MCs) is observable by imaging and electrophysiology. **(A–D)** Electrophysiology. **(A)** Experimental scheme; whole-cell patch-clamp recording was performed in mice anesthetized with ketamine/xylazine. Examples of reconstructed TC (top) and MC (bottom) morphology (from Fukunaga et al., 2012). **(B)** Top left: average membrane potential triggered by inhalation onset, for example, TC. The dotted line represents the start of inhalation. Action potentials (APs) had been clipped. Bottom left: raster plot of AP occurrences for a 700 ms window from the inhalation onset for the same example TC as the top panel. Right: the same but for an example MC. **(C)** Peristimulus time histogram of APs for all morphologically identified TCs (left) and MCs (right). The histogram height is normalized by the number of inhalation onsets, and bin size = 20 ms. **(D)** Top: warped subthreshold Vm for example TC (blue) and example MC (red) in polar coordinates. Mean ± SEM shown. Arrows indicate the resultant vectors for the example TC (blue) and MC (red). Middle and bottom: distribution of resultant vector directions for all morphologically identified TCs (blue) and MCs (red) for subthreshold Vm (middle) and AP histogram (bottom). **(E–I)** Imaging from M/TCs. **(E)** Experimental scheme; two-photon imaging of GCaMP6f in TCs and MCs from anesthetized mice. Middle and bottom: example field of view showing tufted cells (middle) and mitral cells (bottom). Scale bar = 100 μm and 50 μm for top and bottom. **(F)** Left: inhalation-triggered averages from all TCs that couple significantly to sniff for anesthetized mice; the fluorescence fluctuation is normalized in amplitude and shown with grayscale, and ROI index sorted by the time of peak. Right: same but for MCs. **(G)** Left: distribution of peak times for inhalation-triggered average for TCs. *N* = 863 ROIs, 15 mice. Right: same, but for MCs. *N* = 315 ROIs. **(H)** Average fluorescence transients when “warped” and shown concerning the phase of the sniff cycle in a polar plot. Top: normalized waveform for an example TC (blue) and an example MC (red), with corresponding resultant vector. Bottom: averages of normalized waveforms from all significantly coupled TCs (blue) and MCs (red). **(I)** Distribution of resultant vector directions plotted as a polar histogram. Tick marks correspond to proportions of ROIs.

While inputs to the OB arrive locked to sniff rhythms, previous studies reported that the sniff-locking diverges in mitral and tufted cells (Fukunaga et al., 2012; Igarashi et al., 2012; Phillips et al., 2012; Jordan et al., 2018a). These studies observed sniff-locking of OB output neurons in electrophysiological measures. Over the past years, fluorescence imaging has become a standard method to study neuronal activity *in vivo* (Tian et al., 2009; Yang and Yuste, 2017). Many imaging methods still rely on calcium ions and their indicators, which are known to have different dynamics (Akerboom et al., 2012; Chen et al., 2013). It is unclear how the baseline sniff-locking compares when observation methods differ, namely between sub- and suprathreshold membrane potentials from electrophysiology (Figure 2A) and calcium signals obtained by GCaMP6f imaging (Figure 2E). We aimed to establish this relationship first in anesthetized animals, where the sniff rhythm is more regular. As before, we examined the latency from inhalation onset in absolute time and second concerning the phase of the sniff cycle, with the same significance criteria as above.

Electrophysiologically, in anesthetized animals, the membrane potential among morphologically identified TCs peaks 212 ± 14.9 ms after the onset of inhalation. MCs, on the other hand, show peaks immediately after the inhalation onset (Figure 2B; 120.6 ± 45.5 ms since inhalation onset), possibly reflecting depolarizations driven by the previous inhalation. When considering only the depolarization that starts after the inhalation onset, MCs peaked later than TCs, at 404.3 ± 11.7 ms after the onset (*p* < 0.01, unpaired *t*-test for equal means; *n* = 7 cells each for MCs and TCs). This shift in latency is reflected also in the action potential histograms (Figure 2C) and also when expressed with respect to sniff phase [Figure 2D; 95% confidence intervals for resultant vectors = (2.75–5.63) radians for TCs and (0.83–2.66) radians for MCs].

To assess how sniff-locking of MCs and TCs appears when a calcium indicator is used, two-photon imaging of MCs and TCs was performed in anesthetized animals that express GCaMP6f in Tbx21-expressing neurons [Tbet-cre:Rosa-GCaMP6f; (Haddad et al., 2013; Madisen et al., 2015)]. Of 3,048 cells observed in 14 animals, 1,278 were TCs, and 1,770 were MCs, based on soma location and morphology (Figure 2E). Inhalation-triggered averages showed robust and significant locking in 863 TCs and 315 MCs (67.5% of TCs and 17.8% MCs; Figure 2F). Among TCs, the peak time of inhalation-triggered average Ca signals has a clear mode, with an average at 280.1 ± 3.5 ms after the inhalation onset (Figures 2A,H). When compared to the peak times of membrane potential and action potentials, this amounts to average delays of 70 ms and 80 ms, respectively (*p* = 0.078 and *p* = 0.055 relative to subthreshold Vm and AP, respectively; unpaired *t*-test for equal means; *n* = 7 cells for electrophysiology). In MCs, however, peaks of Ca signal are distributed more broadly without a clear mode (Figures 2G,H). The difference in sniff-coupling between TCs and MCs is evident also when the data is expressed as the preferred sniff phase by warping the Ca signals to sniff cycles (Figure 2I). Overall, baseline sniff-coupling is most clearly observed in TCs with imaging, though with a slight delay relative to electrophysiology, while the observed coupling is less pronounced in MCs.

### Olfactory Bulb Projection Neurons Exhibit Diverging Sniff-Locking in Awake Animals

Despite a significant change in sniff patterns, OB neurons have been reported to lock to this rhythm even in awake mice (Cury and Uchida, 2010; Shusterman et al., 2011; Fukunaga et al., 2012; Jordan et al., 2018a). However, with changes in the parameters of airflow, and a resulting change in the dynamics of the signals arriving in the OSN as described above, it is unclear what consequences this has for the MC and TC physiology.

**Figure 3:**
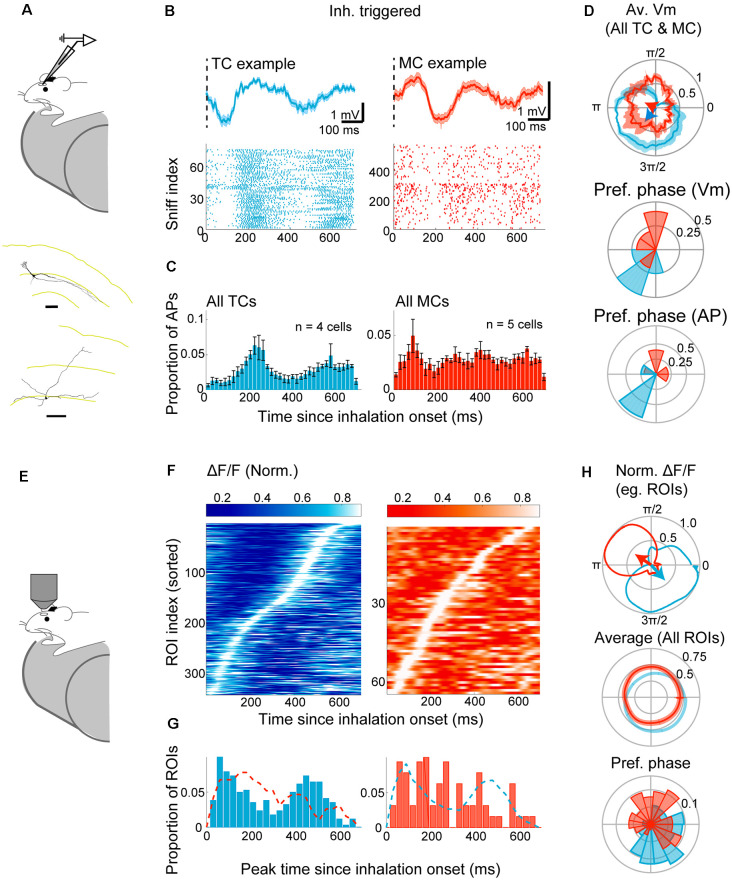
Divergent sniff-coupling is present in awake animals. **(A–D)** Electrophysiology. **(A)** The whole-cell patch-clamp recording was performed in awake mice habituated to head-fixation. Examples of reconstructed TC (top) and MC (bottom) morphology (from Jordan et al., 2018a). **(B)** Top left: inhalation-triggered average Vm for example TC. The dotted line represents the start of inhalation. Bottom left: raster plot of APs in the 700 ms window from the inhalation onset for the same example TC. Right: the same but for an example MC. **(C)** Peristimulus time histogram of APs for all morphologically identified TCs (left) and MCs (right) with histogram height normalized by the number of inhalation onsets. **(D)** Top: Subthreshold Vm for example TC (blue) and example MC (red) expressed as warped average on the polar coordinates. Mean ± SEM shown, with corresponding resultant vectors (arrows). Middle and bottom: distribution of resultant vector directions for all morphologically identified TCs (blue) and MCs (red) for subthreshold Vm (middle) and AP histogram (bottom). **(E–I)** Imaging from M/TCs. **(E)** Two-photon imaging of GCaMP6f in TCs and MCs from awake mice. **(F)** Left: inhalation-triggered averages from all TCs that couple significantly to the sniff cycle for TCs; fluorescence amplitude range normalized to 0–1, and ROI index sorted by the time of peak. Right: same but for MCs. **(G)** Left: distribution of peak times for all TCs. *N* = 341 ROIs, eight mice. Right: same, but for MCs. *N* = 64 ROIs, four mice. **(H)** Average “warped” fluorescence transients in a polar plot. Top: normalized waveform for example TC (blue) and an example MC (red), with corresponding resultant vectors. Bottom: averages of normalized waveforms from all significantly coupled TCs (blue) and MCs (red). **(I)** Polar histogram of resultant vector directions. Tick marks correspond to proportions of ROIs.

To assess this, we re-analyzed previously reported whole-cell patch-clamp recordings from head-fixed awake mice (Figure 3A; Jordan et al., 2018a). This comprised of nine morphologically identified principal neurons (four TCs and five MCs). Analyzing inhalation-triggered waveforms from periods without odors, morphologically identified TCs show a clear peak both in the subthreshold Vm (Figure 3B) and in the AP histograms (Figure 3C), at 238.5 ± 9.0 ms and 210.0 ± 12.9 ms, respectively. Similar to the anesthetized case, MCs show an early peak (134 ± 18.2 ms after the inhalation onset for subthreshold Vm and 95 ± 30.0 ms for AP histogram), as well as a secondary peak with a latency of 356 ± 107.0 ms (290.0 ± 105.4 ms for AP histogram). Concerning the phase of the sniff cycle, TC and MC depolarizations coincide with exhalation and inhalation phases, respectively [Figure 3D; median = 4.0 radians with 95% confidence interval = (3.39, 4.32) radians for TCs, median = 1.9 radians with 95% confidence interval = (1.71 2.91) radians for MCs; average sniff cycle = 379.2 ± 28.7 ms].

To assess how calcium signals compare with the above result, the fluorescence signal was measured from 495 TCs and 357 MCs from eight Tbet-cre:Rosa-GCaMP6f mice (Figure 3E). Of these, the majority of TCs (68.9%) lock significantly to the sniff rhythms (Figure 3F), as measured by the peak amplitude of inhalation-triggered average. As with the anesthetized case, only a small proportion of MCs (17.9%) lock significantly to sniffs, with a more dispersed distribution of peak latencies (Figure 3G). The difference between MCs and TCs is apparent also when analyzed using the sniff cycle phase [95% confidence interval for preferred phase = (5.19–5.58) radians for TCs and (0.42–2.07) radians for MCs; Figure 3H]. On average, the calcium signal is delayed in a manner that is similar to the anesthetized data above average peak time difference = 102 ms relative to AP histogram and 74 (ms relative to subthreshold membrane potential). Overall, our results indicate that distinct locking is preserved in awake animals and can be observed with imaging, though less robustly in mitral cells.

### Reliable Prediction of Cell Type Based on Sniff-Locking Activity

**Figure 4:**
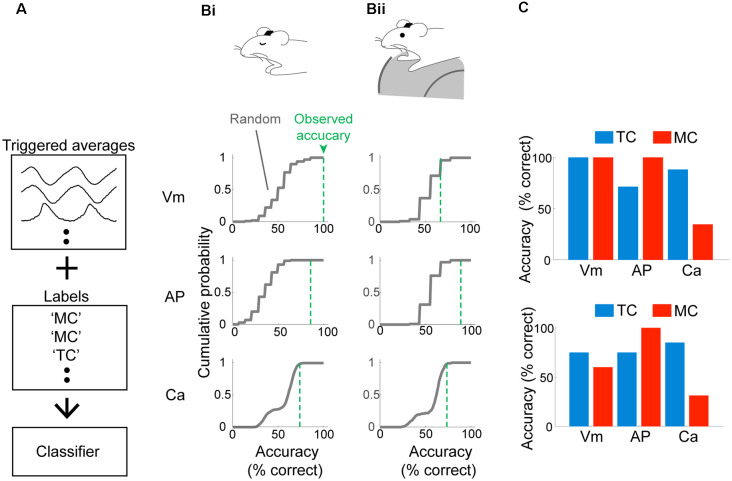
Mitral and tufted cells can be discriminated reliably based on baseline sniff-coupling. **(A)** Approach for classification: inhalation-triggered averages of subthreshold Vm signals were extracted from morphologically identified TCs and MCs. A linear classifier (Fisher discriminant classifier) was constructed based on these and the cell type (“Labels”), such that a discriminant direction maximizes the across-group variance against within-group variance. Two additional classifiers are constructed for inhalation-triggered AP histograms and Ca transients. **(B)** Prediction accuracy for test data: classifier was constructed based on all but one cell, and the predicted identity of the test data was compared against the true identity and repeated for all cells to obtain accuracy (% correct; green dotted lines). The random performance was evaluated by predicting the identity of randomly aligned traces, repeated 100 times to obtain the gray curve. Classifiers were constructed and tested separately for anesthetized **(Bi)** and awake **(Bii)** data. **(C)** Dependence of classifier accuracy on the identity of the neurons.

So far, our analyses focused on morphologically identified MCs and TCs. However, the distinct locking patterns, especially when measured with electrophysiology, raise the possibility that cell types can be determined from physiological signatures alone. To this end, we constructed three Fisher discriminant classifiers (see “Materials and Methods” section; Fisher, 1936) using data from morphologically identified neurons. The three classifiers are to distinguish MCs vs. TCs based on: (1) subthreshold membrane potential; (2) AP patterns; and (3) calcium signal. The performance of the classifiers was validated using a leave-one-out test, where training data comprised average waveforms from all but one cell. This remaining data was used to test the prediction accuracy (Figure 4A). This simple classifier can distinguish between MCs and TCs in the anesthetized case (100% for Vm, 85.7% for AP discharge and 73.9% for calcium signal; *n* = 14 morphologically identified cells; Figure 4Bi). This accuracy exceeded that for random datasets, where the average waveforms were generated by aligning to random time points in the recording. Classifiers for the awake data were generated separately using data from awake animals. Here, the accuracy is generally somewhat lower [66.7% for Vm, 88.9% for APs, and 73.7% for calcium signal (Figure 4Bii)] but still substantially and significantly above chance. In conclusion, the identity of OB output neurons can be accurately estimated based on their baseline sniff-locking activity (Figure 4C).

### Classification Analysis Reveals Distinct Odor-Evoked Responses of Putative MCs and Putative TCs

**Figure 5:**
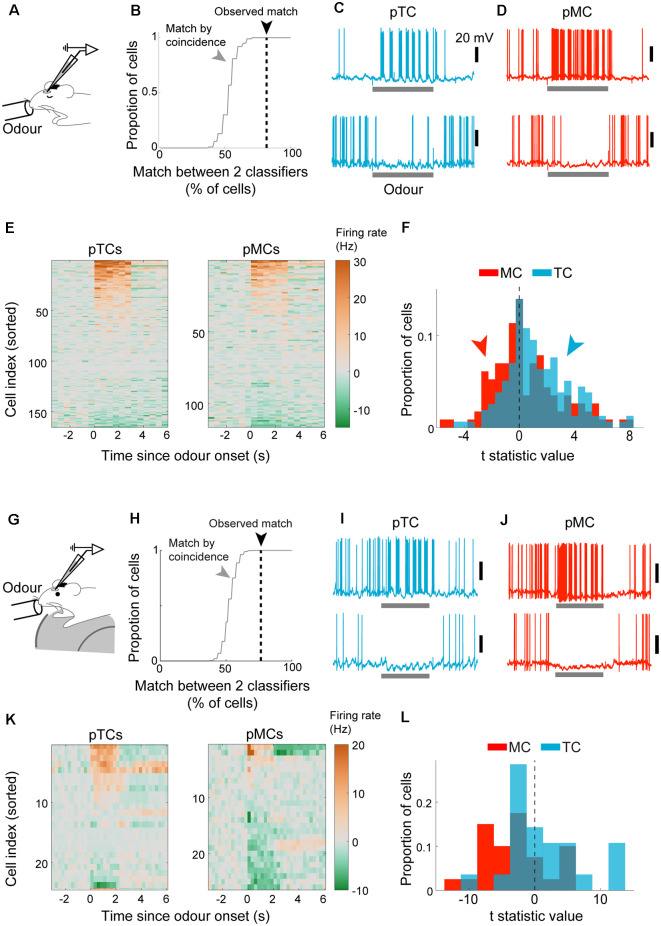
Cell type-specific analysis of evoked responses reveals excitability difference and amplification of the difference in awake animals. **(A–F)** Analysis of evoked responses from anesthetized mice. **(A)** Whole-cell patch-clamp recordings from OB output neurons in anesthetized mice with odor presentations. **(B)** Predicted cell types from the Vm-based classifier and AP-based classifier were compared. Consistency expressed as % of cells where the prediction matched (dotted black line). Random matches were obtained by shuffling the cell orders using random permutation and repeated 100 times to obtain the distribution (gray line). **(C)** Example excitatory response (top) and inhibitory response (bottom) to odors from putative tufted cells. The odor presentation was for 2 s. **(D)** Same as **(C)** but for mitral cells. **(E)** Overview of evoked responses; the average number of action potentials per 500 ms bin was converted into Hz and displayed as a color map. The cell index was sorted according to the mean evoked firing rate. *N* = 165 tufted cell-odor pairs (left) and 116 mitral cell-odour pairs (right). **(F)** Statistics of evoked firing rates; average firing rate during 2-s odor presentation was compared against that during the baseline period (2 s before odor onset) and expressed as a t-statistic; histograms of t-statistics for putative TCs (blue bars) and putative MCs (red bars). **(G,L)** as **(A–F)** but for awake animals. **(G)** Analysis of whole-cell patch-clamp recordings from awake mice habituated to head-fixation and odor presentations. **(H)** The plot of classification consistency as in **(B)** but for awake data. **(I)** Example excitatory response (top) and inhibitory response (bottom) in a putative TC. Scale bar = 20 mV. **(J)** Same as **(I)** but for putative MCs. **(K)** Overview of firing rates around the time of odor presentation (2 s), with cell index, sorted according to the amplitude of evoked responses. **(L)** Distribution of evoked response amplitude as in **(F)**.

Our demonstration above indicates that a linear classifier can reliably assign OB output neurons as MCs or TCs based on their baseline sniff-locking activity. We wished to apply this classification method to whole-cell patch-clamp data from putative M/TCs where no morphological data is available for identification (Figure 5A). We refer to these as “blind” recordings for convenience. Inhalation-triggered averages of Vm or AP histograms were obtained and used to predict the cell type using the Fisher discriminant classifiers generated from morphologically identified cells. Of the 55 cells from anesthetized mice, 33 cells were classified as putative TCs (pTCs) and 22 as putative MCs (pMCs). To validate the classification performance, we compared how well results for Vm-based classification and AP-based classification match. Comparison of the predicted classes indicated that the two classifiers match in 82% of the cases for anesthetized and 76% cases for awake animals, significantly above chance (Figures 5B,H; above 100/100 shuffled control for anesthetized and awake cases).

This high consistency in classification led us to compare how pMCs and pTCs respond to odors (Figures 5C,D). 165 cell-odor pairs for pTCs and 116 cell-odor pairs for pMCs were analyzed from anesthetized mice. To get an overview, APs were counted in 500 ms bins and converted to firing rate (in Hz) as a color map (Figure 5E). Also, to assess if evoked firing rates deviated from baseline firing rates, the average firing rate during 2 s of odor presentation was compared to that just before the odor presentation and expressed as a *t*-statistic (Figure 5F). This revealed that pTCs tend to show more excitatory responses to odors compared to pMCs, both in anesthetized (*p* = 0.007, two-sampled KS test on *t-standardized* firing rates) and awake (*p* = 0.02, *n* = 27 MC-odor pairs vs. 24 TC-odor pairs, two-sample KS test; Figures 5G,I–K) cases. This is consistent with previous data showing greater excitability in TCs relative to MCs (Nagayama et al., 2004; Fukunaga et al., 2012; Burton and Urban, 2014; Livneh et al., 2014; Jordan et al., 2018a). Notably, both the proportion and the magnitude of inhibitory responses were larger in awake mice, in particular for pMCs (Figure 5L; *p* < 0.01, two-sample KS test).

Taken together, this demonstrates that electrophysiological recordings, even where no morphological data is available, can be classified well above chance, though not perfectly, based on baseline sniff-coupling to reveal distinct olfactory representations by MCs and TCs, and further, to reveal state-dependent differences.

### Odor-Evoked Phase Shift in Putative MCs and Inhalation-Linked Hyperpolarization in Putative TCs

In addition to the overall firing rates, MCs and TCs are known to differ in fine temporal patterns during excitatory evoked responses, where TCs modulate firing rates while MCs use phase advance (Fukunaga et al., 2012; Igarashi et al., 2012). This is thought to arise from a greater inhibition experienced by MCs in the absence of odor (Fukunaga et al., 2012, 2014), which in turn can be overcome when excitatory olfactory inputs arrive (Fukunaga et al., 2012).

**Figure 6:**
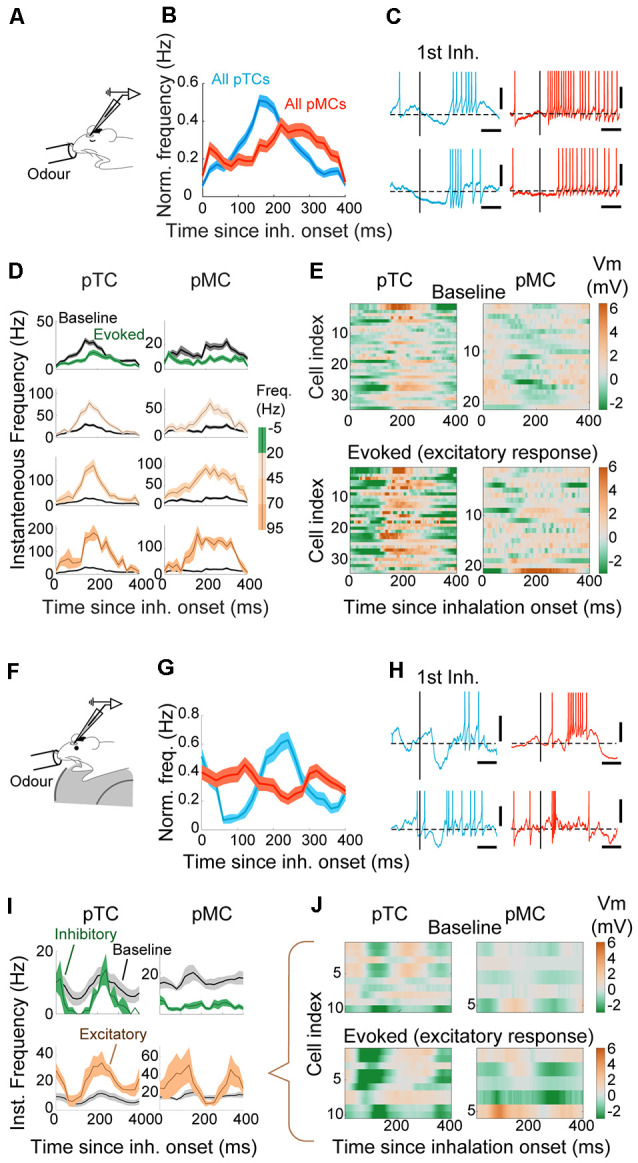
Early-onset hyperpolarization constrains the timing of responses in putative TCs. ** (A)** Whole-cell patch-clamp recordings from anesthetized mice. **(B)** AP histogram is relative to the inhalation onset for all putative TCs (pTCs; blue) and pMCs (red) during odor. **(C)** Examples of excitatory responses for TC (left) and MC (right), on a fine time scale. The vertical bar represents the onset of the first inhalation after odor onset. Scale bar = 100 ms and 10 mV. The horizontal dotted line is the average Vm before the odor. **(D)** AP histogram is relative to the inhalation onset. Responses are grouped by the mean evoked firing rate during odor as indicated on the right. Averages of 88, 47, 15, and 10 TCs and 74, 20, 16, and 4 MCs for respective groups. **(E)** Average Vm relative to the inhalation onset during baseline (top) and excitatory responses (bottom) for putative TCs (left) and putative MCs (right). The average Vm from the baseline period has been subtracted. The amplitude of evoked responses shown as a color map. **(F)** Whole-cell patch-clamp recordings from awake mice. **(G)** Same as **(B)** but for awake data. **(H)** Examples of excitatory responses for tufted (blue) left and mitral (right) cells. AP histogram is relative to the inhalation onset, grouped by the evoked firing rate. **(I)** AP histogram relative to the inhalation onset expressed as Hz, for excitatory (brown) and inhibitory (green) responses (*n* = 10 and 2 cell-odor pairs for pTCs and 5 and 13 cell-odor pairs for pMCs). The baseline AP histogram is shown in gray. **(J)** Mean Vm for cell-odor pairs with excitatory responses relative to the inhalation onset for putative TCs (left) and for putative MCs (right), with mean Vm during baseline period subtracted.

Since only a small number of morphologically identified neurons were included in the previous study, we applied the classifier analysis so that larger, “blind” datasets can be analyzed on this time scale (Figures 6A–C). After classification, for each cell type, data were grouped based on the mean evoked firing rate and plotted as peristimulus time histogram from the onset of inhalation (Figure 6D). In anesthetized mice, during excitatory responses, pTCs generated APs at a largely similar time to the baseline timing (peak time = 160 ± 9.4 ms vs. 150 ± 14.1 ms since inhalation onset, during odor and before odor periods, respectively; *p* = 0.48, paired *t*-test for equal means; *n* = 34 cell-odor pairs). On the other hand, pMCs advanced APs in a graded manner such that APs were observed earlier for responses with greater average evoked firing rates (peak time = 206.7 ± 13.7 ms vs. 268.6 ± 11.9 ms since inhalation onset for excitatory odors and before odor periods, respectively; *p* = 0.002, paired *t*-test for equal means; *n* = 21 cell-odor pairs). Remarkably, odor-evoked APs in some pMCs preceded that of pTCs, indicating that MCs may be able to respond even earlier to odor than TCs, particularly in awake mice (Figures 6F–J). When comparing the odor-evoked membrane potentials of pMCs and pTCs, we found that pTCs consistently exhibit a marked hyperpolarization immediately after the inhalation, both in awake and anesthetized mice (Figures 6E,J). This hyperpolarization is not observed consistently among pMCs (Figures 6E,J). Thus, our results indicate that MCs use a wide range of timing to represent odors, while inhibitory mechanisms likely operate to constrain the timing of TC action potentials during excitatory responses and this difference is accentuated in awake mice.

## Discussion

Sensory representations in the brain often closely reflect the properties of the incoming signals. Understanding this despite changes in conditions, both in the animal’s state, as well as methods of observation, inevitably requires direct comparisons. Here, we investigated how early stages of olfactory processing are shaped by the sniff rhythm in anesthetized and awake mice using electrophysiology and imaging. We show that the nature of sniff-locking can be observed even with imaging at the single-cell resolution, but with a temporal shift relative to electrophysiology, and across states. We demonstrate that a simple linear classifier can distinguish MCs vs. TCs based on their sniff-locking and use it to reveal that the latter experience early-onset hyperpolarization, which may be a mechanism to constrain the timing of excitatory responses. Finally, we show that the difference in the properties of odor encoding between MCs and TCs is accentuated in awake animals.

### Baseline Sniff-Locking Across Methods and States

Our goal was to apply the same sniff measurement and analysis to relate sniff-locking under different methods and brain states. When measured with whole-cell patch-clamp recordings in neurons with average firing rates of 0–10 Hz, sniff-locked activations range over amplitudes of about 10 mV (Cang and Isaacson, 2003; Schaefer and Margrie, 2007; Fukunaga et al., 2012; Kollo et al., 2014; Jordan et al., 2018a,b). This is a moderate range of modulation, but as a previous calcium imaging study reported with population averages (Iwata et al., 2017), we found that sniff-locking is present in the GCaMP6f signal, even at the single-cell level. We observe important differences, however. As expected from different kinetics (Akerboom et al., 2012; Chen et al., 2013), a slight delay was observed in the Ca signal, by 75–100 ms relative to the AP firing measured by patch recordings. This fits with the kinetics of GCaMP6f which was reported to have a rise time of ~50 ms and a decay time of ~150 ms in Layer 2/3 pyramidal neurons in the mouse visual cortex V1 *in vivo* (Chen et al., 2013).

Consistent with previous studies (Carey et al., 2009; Iwata et al., 2017; Moran et al., 2019), we found that OSN input signals lock to sniff rhythms, both in anesthetized and awake animals. We observed a modest increase in latency to peak in awake mice. At first glance, this may seem inconsistent with a previous study (Iwata et al., 2017), which described a phase advance with increased frequency of artificially-induced nasal airflow. However, the change in Ca dynamics in our hands may be due to an additional change in air velocity, or a possible modulatory influence that accompanies changes in the brain state. The difference in the baseline sniff-locking, as well as in the responses to odors, that exists between the two brain states may be due both to the dynamics of airflow, as well as modulatory influences. For example, in a study where the flow of air was experimentally controlled *via* double tracheotomy in anesthetized mice, changes in the frequency of ventilations revealed frequency-dependent filtering properties of the OB output, which were not predicted linearly (Carey and Wachowiak, 2011). Further, a study in awake, behaving mice reported that changes in sniffing frequency cause opposing effects on the two classes of OB output—TCs tend to depolarize with faster sniffs, while MCs tend to hyperpolarize (Jordan et al., 2018b). Thus, changes in the dynamics of airflow may recruit OB circuits in a complex manner. As for the modulatory input, almost all regions that are known to project to the OB and modulate its physiology, including the basal forebrain (Zhan et al., 2013; Rothermel et al., 2014), locus coeruleus (Jiang et al., 1996), and the dorsal raphe nuclei (Petzold et al., 2009), are potential targets of anesthetics (Laalou et al., 2008; McCardle and Gartside, 2012; Vazey and Aston-Jones, 2014). Changes in the level of modulatory inputs may, therefore, explain some of the state-dependent differences described here.

We observed OSN sniff-locking, in general, to be somewhat variable, as a previous study also described (Iwata et al., 2017). In contrast, within the OB, especially for TCs, the peak distribution was markedly sharper. Similarly, compared to the case with OSN terminals, the fraction of sniff-locked OB neurons remains similar in the awake state. While it is beyond the scope of this study to determine the mechanisms, this may reflect additional circuit properties of the OB, such as inhibition, that sharpens the temporal patterns (Margrie and Schaefer, 2003). Indeed, many inhibitory neurons in the OB, including juxtaglomerular (Kato et al., 2013; Miyamichi et al., 2013) and GCs (Cang and Isaacson, 2003; Margrie and Schaefer, 2003; Cazakoff et al., 2014), show sniff-locking themselves (Fukunaga et al., 2014). Future experiments will reveal a more complete circuitry, perhaps dedicated to each stream of information, in refining activity driven by rhythmic inputs.

### Parallel Streams of Olfactory Processing

While the ability to analyze olfactory representations in a cell-type-specific manner is crucial, for some techniques such as electrophysiology *in vivo*, cell-type identification has remained difficult. This is especially the case when relying on morphological reconstruction, which limited sample sizes to date (Fukunaga et al., 2012, 2014; Díaz-quesada et al., 2018; Jordan et al., 2018a). Even though the performance of the linear classifiers is not perfect, the potential to include a far larger sample size for analyses may be advantageous when exploring cell-type-specific olfactory encoding. In our hands, the accuracy of the classifier performance critically depends on the precision of extracted sniff events, which ultimately depends on the quality of recorded sniff waveforms. Also, as many cell types exist in the OB, including small excitatory neurons without lateral dendrites (Hayar et al., 2004; Antal et al., 2006), as well as a large number and variety of inhibitory neurons that spike (Margrie and Schaefer, 2003; Murphy et al., 2005; Pressler and Strowbridge, 2006; Eyre et al., 2008), it is important that some effort is made to restrict the analysis to putative M/TCs, which are large, show prominent after-hyperpolarizations (Kollo et al., 2014) and lock robustly to sniff rhythms at baseline.

A surprising outcome of the wider cell-type-specific analysis was the discovery of early-onset hyperpolarization during excitatory odor responses among TCs. It is possible that constraining the timing of olfactory responses in TCs is more important than previously thought, with hyperpolarization possibly playing a key role to ensure precision (Schaefer et al., 2006). The difference in the way MCs and TCs respond to odors leads to the question of decoding mechanism and computations downstream (Giessel and Datta, 2014). One of the areas that TCs preferentially project to is the olfactory tubercle (Scott et al., 1980). Recordings from this area indicate that only a small fraction (~20%) of neurons here show sniff-locked evoked responses (Payton et al., 2012; Rampin et al., 2012). On the other hand, neurons that show robust sniff-locked responses are common in the piriform cortex (Rennaker et al., 2007; Poo and Isaacson, 2009). However, the majority of recordings to date are from the anterior piriform cortex, which is known to receive projections from both MCs and TCs (Haberly and Price, 1977; Igarashi et al., 2012). While recordings from the posterior piriform cortex remain scarce, more neurons here seem to be sensitive to timing information (Haddad et al., 2013). It will be an intriguing future study to resolve how decoding mechanisms may differ between secondary olfactory areas that participate in parallel information processing, and ultimately, how the two streams of information guide animal behavior.

## Data Availability Statement

The raw data supporting the conclusions of this article will be made available by the authors upon request.

## Ethics Statement

The animal study was reviewed and approved by the Ethics panel of the Francis Crick Institute and the OIST Graduate University.

## Author Contributions

TA, RJ, IF and AS designed the experiments. TA, RJ and IF performed experiments and analyzed the data. TA and IF wrote the manuscript with input from all authors.

## Conflict of Interest

The authors declare that the research was conducted in the absence of any commercial or financial relationships that could be construed as a potential conflict of interest.

## Footnotes

^1^https://github.com/MouseLand/suite2p
