# Supplementary figures and images for: Respiration-Locking of Olfactory Receptor and Projection Neurons in the Mouse Olfactory Bulb and Its Modulation by Brain State
Source: Front Cell Neurosci. 2020 Jul 16;14:220. doi: 10.3389/fncel.2020.00220 (PMC7378796; doi:10.3389/fncel.2020.00220)

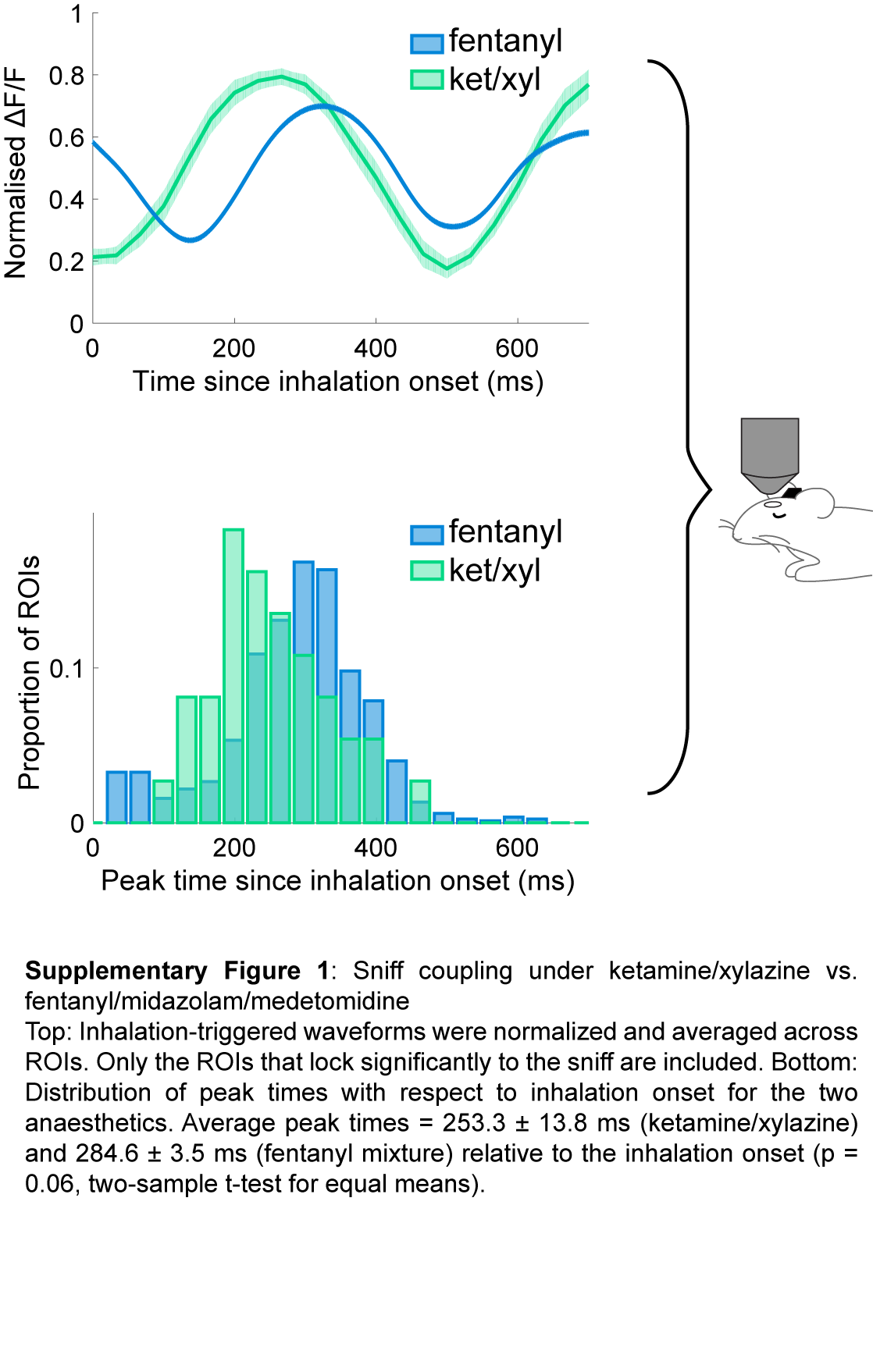

Supplement: Supplementary file 1 [file Image_1.TIF]
